# Occupational Risks during a Monkeypox Outbreak, Wisconsin, 2003

**DOI:** 10.3201/eid1308.061365

**Published:** 2007-08

**Authors:** Donita R. Croft, Mark J. Sotir, Carl J. Williams, James J. Kazmierczak, Mark V. Wegner, Darren Rausch, Mary Beth Graham, Seth L. Foldy, Mat Wolters, Inger K. Damon, Kevin L. Karem, Jeffrey P. Davis

**Affiliations:** * Wisconsin Department of Health and Family Services, Madison, Wisconsin, USA; † Centers for Disease Control and Prevention, Atlanta, Georgia, USA; ‡ Waukesha County Health Department, Waukesha, Wisconsin, USA; § Medical College of Wisconsin, Milwaukee, Wisconsin, USA; ¶ City of Milwaukee Health Department, Milwaukee, Wisconsin, USA; 1 Current affiliation: University of Wisconsin School of Medicine and Public Health, Madison, Wisconsin, USA; 2 Current affiliation: North Carolina Department of Health and Human Services, Raleigh, North Carolina, USA

**Keywords:** occupational diseases, veterinary medicine, monkeypox, domestic animals, commerce, infection control, research

## Abstract

We determined factors associated with occupational transmission in Wisconsin during the 2003 outbreak of prairie dog–associated monkeypox virus infections. Our investigation included active contact surveillance, exposure-related interviews, and a veterinary facility cohort study. We identified 19 confirmed, 5 probable, and 3 suspected cases. Rash, headache, sweats, and fever were reported by >80% of patients. Occupationally transmitted infections occurred in 12 veterinary staff, 2 pet store employees, and 2 animal distributors. The following were associated with illness: working directly with animal care (p = 0.002), being involved in prairie dog examination, caring for an animal within 6 feet of an ill prairie dog (p = 0.03), feeding an ill prairie dog (p = 0.002), and using an antihistamine (p = 0.04). Having never handled an ill prairie dog (p = 0.004) was protective. Veterinary staff used personal protective equipment sporadically. Our findings underscore the importance of standard veterinary infection-control guidelines.

## Abstract 2

Veterinary staff were at high risk; standard veterinary infection-control guidelines should be followed.

During May–June 2003, an outbreak of monkeypox virus (MPXV) infections, initially detected in Wisconsin, occurred in the midwestern United States (*1*,*2*). These MPXV infections were the first to be reported outside of Africa and involved a West African viral strain (*1*,*3*). African rodents imported from Ghana were implicated in virus introduction in the United States (*2*,*4*–*7*). The African rodents had been transported and housed with native prairie dogs that were subsequently distributed as household pets in Wisconsin (*1*). Veterinary and pet store staff are at risk for potentially serious occupationally related infections (*8*–*18*). Early links between MPXV infections and prairie dog exposures in veterinary facilities and pet stores (*1*) led us to investigate occupationally related exposures.

We conducted an outbreak investigation and a veterinary staff cohort study to quantify and characterize all cases that occurred during the 2003 Wisconsin MPXV outbreak, identify protective and risk factors for occupationally transmitted infections, and determine veterinary work practices amenable to infection-control guidelines. Because both investigations were urgent outbreak control measures, no institutional review board approval or written consent was required or obtained.

## Methods

### Outbreak Investigation

The Wisconsin outbreak case definition (Appendix) was similar to case definitions established by the Centers for Disease Control and Prevention (CDC) for human MPXV infection (*19*). Cases were classified as confirmed, probable, or suspected according to clinical, epidemiologic, and laboratory criteria. Case finding was done through electronic postings (email and website postings), faxes, and mass media. Active surveillance of persons in contact with infected persons or animals included self-recorded diaries of signs and symptoms for 21 days postexposure or daily telephone assessments by local health department personnel. Data were summarized at the Wisconsin Division of Public Health (WDPH).

Willing pet store employees were given a standardized questionnaire to assess prairie dog contact and were offered serologic testing. Affected animal distributors were interviewed about work roles and animal care.

### Veterinary Staff Cohort Study

The eligible cohort was defined as all persons, regardless of work roles, employed at any Wisconsin veterinary facility where at least 1 outbreak-associated prairie dog was treated during May 13–27, 2003. Cohort members were defined as those facility employees who participated in the study. Cohort case-patients were defined as cohort members who had laboratory-confirmed MPXV infections, regardless of the presence or absence of specific signs or symptoms. Tissue confirmation required demonstration of MPXV by viral culture, PCR, immunohistochemistry, or electron microscopy. Although cases could not be serologically confirmed by outbreak case definition criteria, cohort members with MPXV infections confirmed by tissue or serologic testing were defined as cohort case-patients. Serologic confirmation required the finding of elevated orthopox immunoglobulin M (IgM) titers in a specimen obtained within 56 days after rash onset or seroconversion in paired acute- and convalescent-phase specimens. The cohort study had no probable or suspected-case definitions and, hence, no probable or suspected cases.

Signs and symptoms surveyed were rash, fever, chills, sweats, headache, joint pain, or lymphadenopathy within 21 days of most recent exposure to an ill prairie dog. Cohort members with a history of vaccinia vaccination or unknown vaccination status and birth date before 1972 were defined as vaccinia-vaccinated.

A standardized questionnaire was used to determine exposure to prairie dogs, general work practices, demographic information, and medical history. Questions to assess contact with prairie dogs during the reception, initial examination, ongoing medical care, and discharge of the prairie dogs had possible answers of yes, no, unknown, or not applicable. Cohort members who did not work within 48 hours after the prairie dog’s veterinary visit were excluded from the exposures analysis but included in the remainder of analyses. Questions about general work practices such as sanitizing, hand hygiene (handwashing or cleaning with alcohol gel), and animal bedding changing practices had possible answers of yes, no, unknown, or not applicable; or they used Likert-scale responses of always, usually, sometimes, rarely, never, or not applicable.

WDPH or local health department personnel administered the confidential questionnaire in person or by telephone. Data were entered into Microsoft Office Access 2003 (Microsoft Corp., Redmond, WA, USA) and analyzed using Epi Info version 3.3 (CDC, Atlanta, GA, USA). Likert-scale responses of always and usually were dichotomized from sometimes, rarely, and never. Responses of unknown or not applicable were excluded.

Willing participants provided acute- and convalescent-phase serum specimens, which were tested for nonspecific orthopox virus IgM and IgG levels at the CDC poxvirus laboratory (*20*). Tissue testing was conducted as part of patients’ clinical care.

Outbreak-associated prairie dogs treated in Wisconsin veterinary facilities were traced backward and forward. Information was obtained about their illnesses and treatments.

## Results

### Outbreak Investigation

**Figure 1:**
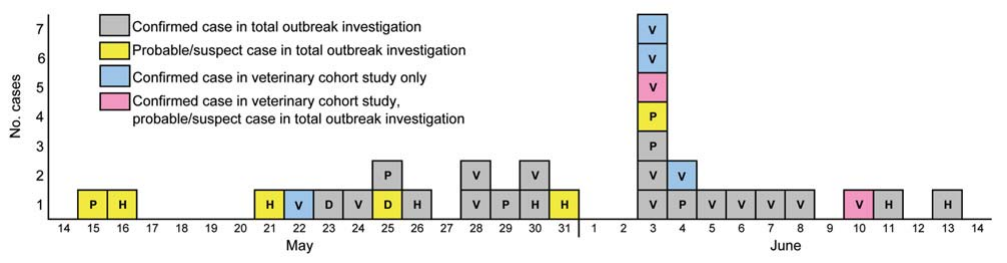
Reported dates of illness onset for persons with monkeypox virus infection. Data from the outbreak investigation and the veterinary facility cohort study, by exposure classification and case status, Wisconsin, 2003. One veterinary cohort case-patient is not included in this figure because of unknown date of illness onset. P, pet store employee or visitor; H, household contact; V, veterinary facility staff; D, animal distributor.

WDPH received 104 reports of potential human MPXV infections. Of these, 27 represented case-defined illnesses: 19 (70%) confirmed, 5 (19%) probable, and 3 (11%) suspected. Illness onsets occurred during May 15–June 13, 2003 (Figure 1). Based on date of first exposure, the median incubation period was 12 days (range 1–41 days). Median age of case-patients was 28 years (range 3–48 years), and 18 (67%) were female. Patients resided in 5 Wisconsin counties: Milwaukee (n = 14), Waukesha (n = 8), Clark (n = 3), Jefferson (n = 1), and Washington (n = 1). Among confirmed case-patients, those positive by test method were distributed as follows: PCR, 15 (79%); immunohistochemistry, 12 (63%); virus culture, 9 (47%); and electron microscopy, 4 (21%).

Signs and symptoms reported by >80% of case-patients were rash, headache, sweats, and fever. Those reported by 60%–70% of case-patients were chills, sore throat, cough, or lymphadenopathy. All other signs and symptoms were reported by <23% of case-patients. No statistically significant differences in signs and symptoms were reported between confirmed and probable or suspected case-patients. Five (19%) patients were hospitalized; none died.

**Figure 2:**
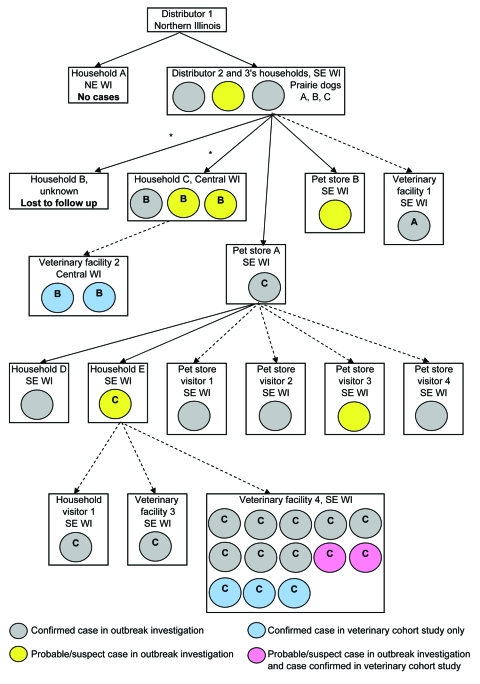
Cases of human monkeypox virus infection. Data from the outbreak investigation and veterinary facility cohort study, by exposure setting and case status, Wisconsin, 2003. A, exposure to prairie dog A; B, exposure to prairie dog B; C, exposure to prairie dog C. Exposure = direct contact or same-room exposure. *Prairie dog sold at swap meet. NE, northeastern; WI, Wisconsin; SE, southeastern; solid arrows, prairie dog sale and exposure; dashed arrows, prairie dog exposure only.

In terms of exposure settings, 12 (44%) cases, including 10 confirmed, occurred in staff of veterinary facilities where ill prairie dogs had received care (Figure 2). Other cases occurred in 6 members of households with prairie dogs, 4 pet store visitors, 2 pet store employees, 2 animal distributors, and 1 visitor to a household with prairie dogs. No known cases occurred in healthcare workers who treated patients or in laboratory workers who handled specimens.

Symptom diaries were completed by 258 persons, including 28 pet store employees and 7 veterinary staff. Local health department personnel monitored 243 other persons by telephone, including 77 veterinary staff. Among 501 persons followed up, 10 (2%) experienced illness; all 10 were veterinary staff.

Two pet stores had received outbreak-associated prairie dogs. Of 28 employees (26 from store A, 2 from store B), 19 (68%) completed the questionnaire: 11 had handled prairie dogs, 9 had fed prairie dogs, 2 had been bitten by a prairie dog, and 2 had been scratched by a prairie dog. One store A employee had a confirmed case, and 1 store B employee had a suspected case; both had handled ill prairie dogs. The store A employee had a tissue diagnosis of MPXV confirmed by viral culture and PCR and was positive for orthopox IgM and IgG antibodies in acute- and convalescent-phase serum specimens. The store B employee had prior vaccinia vaccination; a convalescent-phase serum specimen was negative for orthopox IgM and positive for IgG. Approximately 2 months after the last prairie dog exposure, serum specimens were obtained from 12 noncase pet store employees, 4 of whom had handled ill prairie dogs. All 12 had negative orthopox IgM antibody results.

Two Wisconsin exotic animal distributors (distributors 2 and 3, a married couple) distributed outbreak-associated prairie dogs and housed animals in their home. Distributor 2 had a confirmed case of MPXV infection, distributor 3 had a suspected case, and an immunocompromised household member who had no direct animal contact had a confirmed case.

### Veterinary Staff Cohort Study

Four veterinary facilities had treated 3 outbreak-associated prairie dogs. These facilities employed 81 (range 3–59) persons during the outbreak; 74 (91%) participated in the cohort study (cohort members), and 44 (54%) participated in the serosurvey. Table 1 summarizes demographic characteristics of cohort members, serosurvey participants, and cohort case-patients. At least 1 veterinarian from each facility was a cohort case-patient. Among 17 cohort case-patients, 8 (47%) had tissue and serologically confirmed cases, 7 (41%) had serologic confirmation only, and 2 (12%) had tissue confirmation only. The 17 cohort case-patients included all 10 veterinary staff with confirmed cases and 2 with probable or suspected cases (previously mentioned in the overall outbreak investigation). Five serologically confirmed cohort case-patients did not meet the definition of an overall outbreak case-patient.

**Table 1:** Demographic characteristics of veterinary facility cohort members during outbreak of monkeypox virus infections, Wisconsin, 2003

Demographic variable*	All cohort members (n = 74), no. (%)	Serosurvey participants only (n = 44), no. (%)	Cohort case-patients only (n = 17), no. (%)
Sex			
Female	56 (76)	34 (77)	13 (76)
Male	18 (24)	10 (23)	4 (24)
Race			
White	73 (99)	44 (100)	17 (100)
Unknown	1 (1)	0	0
Ethnicity			
Not Hispanic	67 (90)	40 (91)	16 (94)
Hispanic	6 (8)	4 (9)	1 (6)
Unknown	1 (1)	0	0
Job title			
Veterinarian	12 (23)	9 (20)	6 (35)
Veterinary technician	18 (24)	14 (32)	6 (35)
Veterinary assistant	18 (24)	11 (25)	5 (29)
Receptionist	14 (19)	6 (14)	0)
Clinic manager	4 (5)	4 (9)	0
Other†	3 (4)	0	0
No. employees			
Facility 1 (n = 4)	4 (5)	3 (7)	1 (6)
Facility 2 (n = 3)	3 (4)	3 (7)	2 (12)
Facility 3 (n = 15)	14 (19)	5 (11)	1 (6)
Facility 4 (n = 59)	53 (72)	33 (75)	13 (76)

*Ages, median (range), y: all cohort members, 31 (17–57); serosurvey participants, 31 (20–57); cohort case-patients, 31 (23–47). †Custodial and information services staff.

Fever, sweats, chills, rash, lymphadenopathy, and headache were each associated (p<0.001) with confirmed MPXV infection (Table 2). Among cohort case-patients, 15 (88%) had multiple signs and symptoms and 2 (12%) had only 1 sign or symptom (headache and a nonvesicular, nonpustular rash of unknown onset date, respectively). Severe keratitis required corneal transplantation for 1 cohort case-patient, and a miscarriage occurred at 12 weeks of gestation. Two cohort case-patients did not experience a rash.

**Table 2:** Medical data for veterinary facility cohort members during outbreak of monkeypox virus infections, by case status, Wisconsin, 2003*

Patient data	Cohort case-patients, no./total† (%)	Cohort members without confirmed case, no./total† (%)	p value‡	RR	95% CI
Signs and symptoms					
Rash	15/17 (88)	3/57 (5)	<0.001	23.3	5.9–92.4
Fever	12/16 (75)	5/57 (9)	<0.001	9.9	3.7–26.7
Chills	14/17 (82)	4/57 (7)	<0.001	14.5	4.7–44.9
Sweats	14/17 (82)	8/57 (14)	<0.001	11.0	3.5–34.6
Headache	13/17 (76)	7/57 (12)	<0.001	8.8	3.2– 23.8
Joint pain	6/17 (35)	7/56 (13)	0.06	2.5	1.1–5.6
Lymphadenopathy	11/16 (69)	2/57 (4)	<0.001	10.2	4.3–24.3

Exposures to ill prairie dogs					
Was in clinic within 48 h after prairie dog visit	17/17 (100)	50/57 (88)	0.19	UND	NA
Admitted prairie dog to facility	1/17 (6)	6/44 (14)	0.66	0.5	0.1–3.1
Involved in initial evaluation of prairie dog	5/16 (31)	1/44 (2)	0.004	4.1	2.2–7.7
Cared for animal within 6 feet of prairie dog	9/12 (75)	12/31 (39)	0.03§	3.1	1.0–10.0
Gave oral antimicrobial drugs	3/12 (25)	4/40 (10)	0.33	2.1	0.8–6.0
Gave subcutaneous fluids	4/12 (33)	5/41 (12)	0.18	2.4	0.9–6.4
Gave subcutaneous antimicrobial drugs	1/11 (9)	3/41 (7)	1.00	1.2	0.2–7.2
Gave nebulized therapy	3/12 (25)	4/41 (10)	0.18	2.2	0.8–6.2
Involved in follow-up examination	5/12 (42)	5/41 (12)	0.04	3.1	1.2–7.7
Took radiographs of prairie dog	1/11 (9)	1/41 (2)	0.38	2.5	0.6–11.1
Fed prairie dog	4/12 (33)	0/41 (0)	0.002	6.1	3.3–11.6
Cleaned cage of prairie dog	12/12 (100)	36/43 (84)	0.32	UND	NA
Cleaned discharge from prairie dog's eyes	6/15 (40)	7/41 (17)	0.09	2.2	1.0–5.0
Examined any animal on surface used to examine prairie dog within past 24 h	7/11 (64)	9/30 (30)	0.07	2.7	1.0–7.9
Spent >30 min handling prairie dog	4/17 (24)	4/53 (8)	0.09	2.4	1.0–5.6
Never handled prairie dog	7/17 (41)	43/53 (81)	0.004	0.28	0.1–0.6
Spent >30 min within 3 feet of prairie dog	7/17 (41)	14/53 (26)	0.25§	1.6	0.7–3.7
Job involved direct animal care	17/17 (100)	36/57 (63)	0.002	UND	NA

Medical history					
Vaccinia vaccination	7/17 (41)	23/57 (40)	0.95§	1.0	0.4–2.4
Atopic dermatitis	1/17 (6)	4/57 (7)	1.0	0.9	0.1–5.2
Seasonal allergies	7/17 (41)	21/57 (37)	0.75§	1.2	0.5–2.7
Open sores at time of prairie dog visit	5/17 (29)	9/57 (16)	0.28	1.8	0.8–4.3
Upper respiratory infection at time of prairie dog visit	3/17 (18)	3/57 (5)	0.13	2.4	1.0–6.1
Antihistamine use at time of prairie dog visit	4/17 (24)	3/57 (5)	0.04	3.0	1.3–6.6

*RR, relative risk; CI, confidence interval; UND, undeterminable; NA, not accurate. †Denominators vary according to total no. persons with work roles appropriate to the exposure; e.g., a receptionist would not be expected to administer subcutaneous fluids. ‡Fisher exact 2-tail test unless otherwise indicated. §Mantel-Haenszel test.

By using the number of cohort members from each facility as the denominator, we calculated veterinary facility attack rates as follows: facility 1, 25%; facility 2, 67%; facility 3, 7%; and facility 4, 25%. The attack rate among cohort members for all 4 facilities combined was 23%. All cohort case-patients had been in the veterinary facility within 48 hours of the prairie dog’s visit. The only factor protective against MPXV infection (Table 2) was never having handled an ill prairie dog (p = 0.004). Having a job involving direct animal care (e.g., veterinarian, veterinary technician, or veterinary assistant) was associated with having a confirmed case (p = 0.002). Four types of exposures were associated with having a confirmed case: participating in an initial (p = 0.004) or follow-up (p = 0.04) examination of an ill prairie dog, caring for an animal within 6 feet of the ill prairie dog (p = 0.03), and feeding the ill prairie dog (p = 0.002). Vaccinia vaccination status did not differ between those who performed at least 1 of these 4 high-risk activities and those who did not (p = 0.9). All cohort case-patients reported having practiced hand hygiene after examining or feeding the ill prairie dog. Gloves had been used by cohort case-patients during the following activities: 2 (40%) initial examination, 3 (60%) follow-up examination, and 3 (75%) feeding an ill prairie dog. No cohort case-patients reported having used surgical masks, goggles, or face shields during these high-risk activities. Four cohort case-patients had fed a prairie dog on 8 occasions: placed food in the cage without touching the prairie dog (1×), hand fed prairie dog by syringe (3×), placed food directly in the prairie dog’s mouth (3×), or fed through gastric tube (1×). Although having spent >30 minutes handling the prairie dog approached significance (p = 0.09), 7 (41%) cohort case-patients reported never having handled a prairie dog. All of these 7 cases resulting from indirect exposure occurred in employees of facility 4. Five of these 7 case-patients reported having been within 3 feet of prairie dog C, 1 reported having been in the same room as prairie dog C but not within 3 feet, and 1 reported not being in facility 4 while prairie dog C was there but being there within 48 hours of its death. Multivariate analysis was not possible because of the small number of cohort members with each type of exposure.

Using antihistamines during the prairie dog visit (p = 0.04) was associated with being a cohort case-patient (Table 2). Antihistamine use was considered a possible surrogate for hand-to-face contact because users of antihistamines generally have allergies or rhinorrhea and likely touch their eyes or nose frequently. No other personal medical history was associated with illness. Previous vaccinia vaccination (p = 0.95) was not protective against MPXV infection. Few cohort members reported immunosuppressive medication use (n = 3), immunosuppressive illness (n = 2), or being pregnant (n = 2).

No general work practice was a protective or risk factor for being a cohort case-patient. General work practices were not outbreak specific and were used to assess overall risk for communicable disease transmission. Several cohort members reported hand-to-mouth activities (eating, drinking, chewing gum, or applying lip products) in animal care areas (Table 3). Only 12% who cleaned ill animals’ cages reported having used gloves during this task. Most (92%–93%) cohort members reported cleaning their hands before eating at work and after ill animal contact.

**Table 3:** General work practices of 74 veterinary facility cohort members during outbreak of monkeypox virus infections, Wisconsin, 2003*

Work practice	No. (%)
Sanitizes examination table	44 (81)
Sanitizes examination room countertops	32 (59)
Eats in work break room	59 (82)
Eats where animals are treated or housed	1 (1)
Drinks where animals are treated or housed	10 (14)
Chews gum where animals are treated or housed	11 (15)
Applies lip products where animals are treated or housed	6 (8)
When cleaning cages, agitates bedding enough to aerosolize material in cage	5 (10)
Wears gloves when cleaning ill animals' cages	6 (12)
Does animal laundry at work	31 (58)
Cleans hands after contact with ill animals	65 (93)
Cleans hands before eating at work	67 (92)
Cleans hands when leaves work	57 (77)
Changes out of work shoes before leaving work	8 (11)
Changes out of work clothes before leaving work	7 (11)
Washes work clothes at home	70 (95)
Washes laboratory coat at home	14 (50)
Washes work clothes between work shifts	67 (93)
Washes laboratory coat between work shifts	15 (50)
*The denominators vary according to the number of cohort members who perform a given task.	

The 44 serosurvey participants included 9 of 10 patients with tissue-confirmed cases and 35 of 64 persons without tissue-confirmed cases (p = 0.04). Cohort members with direct animal care jobs were not more likely (p = 0.19) than those without such jobs to have participated in the serosurvey. MPXV infection was serologically confirmed for 13 (65%) persons who provided paired serum specimens and 2 (10%) who provided only acute-phase serum specimens. No evidence of asymptomatic seroconversion was found. Among serosurvey participants, only feeding a prairie dog was statistically associated (p = 0.02) with having a confirmed case of illness, and no personal medical history factors were associated with illness. A history of vaccinia vaccination was not protective against MPXV infection (p = 0.35). Nineteen (43%) serosurvey participants had been vaccinated. Of these, 6 (32%) had MPXV infection; 5 had multiple signs or symptoms; and 1 had only a nonpustular, nonvesicular rash. Four (67%) of the vaccinia-vaccinated serosurvey participants with confirmed cases had serologic confirmation only (no tissue confirmation was attempted), and 1 (17%) had both tissue and serologic confirmation of illness. Two serosurvey participants (A and B) with confirmed cases and previous vaccinia vaccination had no acute elevation of IgM. Participant A had symptomatic illness confirmed by IgG seroconversion and multiple high-risk exposures, including participation in 2 prairie dog examinations and having provided care to an animal within 6 feet of the prairie dog. Participant A’s IgM levels were not elevated at 15, 36, and 50 days after exposure. Participant B had symptomatic, pathologically confirmed illness and multiple high-risk exposures, including participation in 4 prairie dog examinations, having fed the prairie dog, and having provided care to an animal within 6 feet of the prairie dog. Participant B’s IgM results were not elevated, and IgG results were positive without a boost in titer at 16 and 157 days after exposure. Participant B had a history of bone marrow ablation and an allogenic bone marrow transplant.

An Illinois animal distributor (distributor 1) obtained prairie dogs from a Texas distributor (C. Austin, pers. comm.). During April–May, 2003, distributor 1 housed ≈200 prairie dogs with African rodents that had been purchased on April 21 and subsequently implicated in MPXV introduction (*2*,*6*,*7*). Distributor 2 purchased 39 prairie dogs, including prairie dogs A, B, and C, from distributor 1 and transported them to Wisconsin during April 15–May 17 (*1*,*4*). Prairie dog A remained in the custody of distributor 2 until it was taken to facility 1 (Figure 2) for 10 minutes for carbon dioxide chamber euthanasia on May 13, 2003. Prairie dog B was sold at a swap meet on May 11; became ill with conjunctivitis, lymphadenopathy, and papular skin lesions on May 13; was examined at facility 2 for 10–30 minutes on May 15; and died on May 20. Prairie dog C was sold to pet store A on May 5 and sold to a customer on May 17. On May 19, prairie dog C had illness onset with conjunctivitis, skin lesions, and respiratory disease. Prairie dog C was examined in facility 3 for 20–25 minutes on May 22 and hospitalized at facility 4 from May 25 until its death on May 27. Facility 4 staff provided extensive treatment including repeated examinations; hand feeding; eye discharge removal; and oral, subcutaneous and nebulized treatments. Sixteen (59%) outbreak cases and 14 (82%) veterinary cohort cases were associated with prairie dog C.

## Discussion

The 2003 outbreak of MPXV infections affected Wisconsin residents who had been exposed in multiple settings; however, 59% of cases occurred among occupationally exposed persons. Our cohort study demonstrates that veterinary staff were particularly at risk (23% attack rate). Pet store employees were at lower risk (7% attack rate). Infected prairie dogs were probably more ill and shedding more virus while in veterinary facilities than in pet stores, which would account for more observed infections among veterinary staff. Both Wisconsin distributors of ill prairie dogs became ill. The preponderance of occupationally acquired cases was unique to Wisconsin during this outbreak. Among other involved states, 1 veterinarian from Indiana had a suspected case, and cases occurred in 2 employees of distributor 1 (*7*,*21*,*22*).

Most outbreak cases (59%) and veterinary cohort cases (82%) were associated with exposure to prairie dog C. We found no intrinsic differences in the monkeypox infection of prairie dog C compared with that of prairie dogs A or B and no explanation for this association, although length and type of exposures in facility 4 were likely accountable. Facility 4 had many more employees than the other facilities, and prairie dog C was hospitalized there for a relatively prolonged period (3 days) and received extensive treatments there. In addition, the fact that 7 (54%) facility 4 cohort case-patients reported never having handled prairie dog C indicates that other transmission modes (e.g., fomites, aerosols) could have contributed to that facility’s large number of cases. Nebulization treatments, which prairie dog C received ≈4× at facility 4, could have exposed employees to MPXV. Although nebulization was performed in an enclosed plastic chamber, the nature of the treatment would foster aerosolization, coughing, and possibly mobilization of respiratory secretions with which employees could have unknowingly come into contact.

Although our Wisconsin investigation showed no definitive evidence of human-to-human transmission of MPXV, which concurs with other states’ findings during this multistate outbreak (*6*,*7*,*21*,*23*), such transmission remains a possibility. Because persons who had had no direct contact with ill animals became ill with MPXV infection, person-to-person transmission within veterinary facilities might have occurred. However, because of the lack of personal protective equipment use among the cohort members and the finding of MPXV in ill prairie dog’s urine and feces (*21*), environmental exposure may well account for these cases.

The substantial amount of illness among veterinary staff underscores the importance of infection-control practices in veterinary settings. Cohort case-patients frequently did not use personal protective equipment during high-risk activities (e.g., examining or feeding ill prairie dogs). Furthermore, cohort members reported general work practices that foster hand-to-mouth activities in animal care areas. Few (12%) cohort members reported having used gloves when cleaning ill animals’ cages, a task that can contaminate staff hands with animal dander, urine, and fecal matter. We cannot determine whether infection-control guidelines would have prevented MPXV infections among veterinary staff, but use of personal protective equipment might have limited viral transmission. The National Association of State Public Health Veterinarians recently released the Veterinary Standard Precautions Compendium (*24*), the first guidelines to describe standard infection-control practices for veterinary facilities. Use of these guidelines should be encouraged.

In contrast with results from a previously published study (*25*), results of our cohort study do not support the conclusion that prior vaccinia vaccination protected against MPXV infection in this outbreak. Hammarlund et al. found that 3 (37%) of 8 vaccinia-vaccinated persons in this outbreak had asymptomatic MPXV infections and surmised that they had long-term immunity against MPXV infection (*25*). Our cohort study showed that previous vaccinia vaccination did not protect against MPXV infection; all previously vaccinated serosurvey participants with positive serologic results had at least 1 sign or symptom of MPXV infection. The more systematic inclusion and analysis of exposed persons within our cohort, compared with the cohort-series approach of Hammarlund et al., may account for the difference in this finding (*26*).

Our case definition for the cohort study differed from that of the overall outbreak investigation. Among veterinary staff, the 2 definitions resulted in different case numbers: the outbreak case definition resulted in 10 confirmed and 2 probable or suspected cases; the cohort study case definition resulted in 17 confirmed cases. Serologic results were not a confirming criterion in the outbreak case definition because their results were not validated at the time and because serologic specimens were not systematically gathered throughout the multistate outbreak.

For unknown reasons, 2 cohort case-patients had no elevation of orthopox IgM. Although the IgM response might have been missed, this is unlikely given the timing of specimen collections. Also, previous vaccinia vaccination might have altered the immune response to the MPXV infection. It is also possible that participant B’s past medical history might have affected the immune response to this infection.

Our study has several potential limitations. Although 91% of employees at the 4 affected veterinary facilities participated in the cohort study, only 54% participated in the serosurvey. Persons with tissue-confirmed illness were more likely than persons without such illness to have participated in the serosurvey. These factors might have resulted in an underestimation of overall cases and limited the detection of asymptomatic seroconversion. Recall bias, which might have overestimated the association between prairie dog contact and illness, was likely limited by relatively brief intervals between exposures and data collection. Finally, statistical analysis beyond univariate analysis was limited because of the small number of cohort members involved in each of the high-risk prairie dog exposures.

Our investigation and cohort study demonstrate that occupational exposure, especially among veterinary staff, was a critical factor during this outbreak. This outbreak highlights the importance of standard infection-control guidelines developed for veterinary settings and the need to encourage their use.

## Supplementary Material

Appendix Case Definitions for Human Monkeypox Virus Outbreak, Wisconsin, 2003
