# Supplementary material for: Occupational Risks during a Monkeypox Outbreak, Wisconsin, 2003
Source: Emerg Infect Dis. 2007 Aug;13(8):1150–7. doi: 10.3201/eid1308.061365 (PMC2828073; doi:10.3201/eid1308.061365)
Supplement: Appendix — Case Definitions for Human Monkeypox Virus Outbreak, Wisconsin, 2003 [file 06-1365_app-s1.pdf]

Appendix.

Case Definitions for Human Monkeypox Virus Outbreak, Wisconsin, 2003

Clinical Criteria

- Rash (macular, papular, vesicular, or pustular; generalized or localized; discrete or confluent)
- Fever (subjective or measured temperature ≥99.3°F [≥37.4°C])
- Other signs and symptoms
  - Chills and/or sweats
  - Headache
  - Backache
  - Lymphadenopathy
  - Sore throat
  - Cough
  - Shortness of breath

Epidemiologic Criteria

- Exposure* to an exotic or wild mammalian pet† obtained after April 14, 2003, with clinical signs of illness (e.g., conjunctivitis, respiratory symptoms, and/or rash)

OR

- Exposure* to an exotic or wild mammalian pet† with or without clinical signs of illness that has been in contact with either a mammalian pet‡ or a human with monkeypox

OR

- Exposure§ to a suspect, probable, or confirmed human case-patient

Laboratory Criteria

- Isolation of monkeypox virus in culture
- Demonstration of monkeypox virus DNA by PCR testing of a clinical specimen
- Demonstration of virus morphologically consistent with an orthopoxvirus by electron microscopy in the absence of exposure to another orthopoxvirus
- Demonstration of presence of orthopoxvirus in tissue by using immunohistochemical testing methods in the absence of exposure to another orthopoxvirus

Geographic and Time Criteria

- Wisconsin resident
- Illness onset in May–June 2003

Case Classification

Suspected Case

- Meets 1 of the epidemiologic criteria

AND

- Fever or unexplained rash and ≥2 other signs or symptoms with onset of first sign or symptom ≤21 days after last exposure meeting epidemiologic criteria

Probable Case

- Meets one of the epidemiologic criteria

AND

- Fever with onset of first sign or symptom ≤21 days after last exposure meeting epidemiologic criteria

OR

- If rash is present but type is not described, demonstrates elevated levels of immunoglobulin M (IgM) reactive with orthopox virus 7–56 days after rash onset

Confirmed Case

- Meets 1 of the laboratory criteria

Exclusion criteria

A case was excluded as a suspect or probable monkeypox case if

- An alternative diagnosis could fully explain the illness¶

OR

- The case was reported on the basis of a primary or secondary exposure to a wild or exotic mammalian pet or a human subsequently determined not to have monkeypox, provided other possible epidemiologic exposure criteria are not present

OR

- Rash had not developed within 14 days of onset of signs or symptoms of monkeypox. If a rash did develop after 14 days of onset of signs or symptoms:
  - Case determined to be negative for nonvariola generic orthopoxvirus by PCR testing of a well-sampled rash lesion
  - Case determined to have undetectable IgM 7–56 days after rash onset

*Included living in a household, petting or handling, or visiting a pet holding facility (e.g., pet store, veterinary clinic, pet distributor).

†Included prairie dogs, Gambian giant rats, and rope squirrels. Exposure to other exotic or wild mammalian pets considered on the basis of likelihood of contact with a mammal with monkeypox and compatibility of clinical illness with monkeypox.

‡Included living in a household or originating from the same pet holding facility as another animal with monkeypox.

§Included skin-to-skin or face-to-face contact.

¶Factors considered in assigning alternative diagnoses included the strength of epidemiologic exposure criteria for monkeypox, diagnostic test specificity, and compatibility of the clinical course.
